# Supplementary material for: Complexome profiling of the Chlamydomonas psb28 mutant reveals TEF5 as an early PSII assembly factor
Source: Plant Cell. 2025 Mar 17;37(6):koaf055. doi: 10.1093/plcell/koaf055 (PMC12164590; doi:10.1093/plcell/koaf055)
Supplement: koaf055_Supplementary_Data [file koaf055_supplementary_data.zip › psb28_interactive.zip!/plots/intensity/qualityPlots/peptides/LHCB5_Cre16.g673650.t1.1.html]

### Cre16.g673650.t1.1

Trivial name: LHCB5  
SeqCoverage [median]: 73.00  
Mass: 30.71 kDa  
  
 

| Sequence | MissedCleavage | Mass [Da] | wtIntensity | muIntensity | Score |
| --- | --- | --- | --- | --- | --- |
| DPETVAK | 0 | 758.38 | 86432.00 | 62224.11 | 190.49 |
| ENELLHAR | 0 | 980.50 | 72534.92 | 60994.62 | 182.53 |
| FDSSVFDGLDPLYPGGPFDPLGLADDPEVLQELK | 0 | 3661.77 | 3747.98 | 0.00 | 60.76 |
| GWLGGQGGAADLDK | 0 | 1343.65 | 56258.20 | 141478.21 | 228.28 |
| GWLGGQGGAADLDKWYGPDR | 1 | 2117.99 | 111795.61 | 208024.99 | 168.79 |
| GWLGGQGGAADLDKWYGPDRK | 2 | 2246.09 | 165689.45 | 280299.46 | 170.06 |
| HVADPFGYNLLTVLGAEER | 0 | 2100.06 | 677832.45 | 628242.43 | 222.50 |
| HVADPFGYNLLTVLGAEERTPTL | 1 | 2512.30 | 4058.42 | 0.00 | 150.91 |
| KLFLPSGLYDR | 1 | 1307.72 | 551044.30 | 1157259.99 | 160.72 |
| KLFLPSGLYDRSEIPEYLNGELAGDYGYDPLGLGKDPETVAK | 3 | 4599.31 | 2219.71 | 0.00 | 41.70 |
| LAMVSVLGFAVQSYVTGEGPYANWTK | 0 | 2787.39 | 875.71 | 0.00 | 79.43 |
| LFLPSGLYDR | 0 | 1179.63 | 1597400.10 | 1847185.34 | 185.55 |
| MQIQALFK | 0 | 977.54 | 31393.78 | 0.00 | 127.12 |
| MQIQALFKK | 1 | 1105.63 | 28561.84 | 8275.02 | 124.08 |
| NGTGPAGYSPGIGK | 0 | 1274.63 | 330533.53 | 389678.28 | 249.25 |
| RNGTGPAGYSPGIGK | 1 | 1430.73 | 2838.62 | 9484.63 | 93.24 |
| SEIPEYLNGELAGDYGYDPLGLGK | 0 | 2569.22 | 6475.77 | 0.00 | 71.65 |
| SEIPEYLNGELAGDYGYDPLGLGKDPETVAK | 1 | 3309.59 | 82131.19 | 191468.42 | 140.04 |
| TGASAPAK | 0 | 701.37 | 23090.25 | 45013.83 | 191.63 |
| TGASAPAKK | 1 | 829.47 | 0.00 | 861.73 | 99.14 |
| WAMLAAAGILIPEGLQANGANIK | 0 | 2321.26 | 0.00 | 8179.41 | 123.55 |
| WYGPDRK | 1 | 920.45 | 1686.53 | 5349.36 | 86.87 |
| YRENELLHAR | 1 | 1299.67 | 1572933.84 | 1645838.03 | 192.64 |
|  |  |  | 5409534.2 | 6689857.8 |  |
